# Supplementary material for: Unveiling the Bioactive Potential of the Invasive Jellyfish Phyllorhiza punctata Through Integrative Transcriptomic and Proteomic Analyses
Source: Biomolecules. 2025 Aug 4;15(8):1121. doi: 10.3390/biom15081121 (PMC12383608; doi:10.3390/biom15081121)
Supplement: Supplementary file 1 [file biomolecules-15-01121-s001.zip › Suplementary Material/FileS3/PEPFOLD4/XP_065072245_15793/clusters.html]

 PEP-FOLD clusters


Download archive of all models  here   

|  |  |  |
| --- | --- | --- |
| # | file | sOPEP |
| # cluster 1 - Size: 9 |  |  |
|  | XP\_065072245\_15793-model1 | -14.7851 |
|  | XP\_065072245\_15793-model1.1 | -14.7851 |
|  | XP\_065072245\_15793-model1.2 | -14.7851 |
|  | XP\_065072245\_15793-model1.3 | -14.7851 |
|  | XP\_065072245\_15793-model1.4 | -14.6318 |
|  | XP\_065072245\_15793-model1.5 | -14.6318 |
|  | XP\_065072245\_15793-model1.6 | -14.6318 |
|  | XP\_065072245\_15793-model1.7 | -14.6318 |
|  | XP\_065072245\_15793-model1.8 | -14.6318 |
| # cluster 2 - Size: 8 |  |  |
|  | XP\_065072245\_15793-model2 | -14.714 |
|  | XP\_065072245\_15793-model2.1 | -14.714 |
|  | XP\_065072245\_15793-model2.2 | -14.714 |
|  | XP\_065072245\_15793-model2.3 | -14.714 |
|  | XP\_065072245\_15793-model2.4 | -14.714 |
|  | XP\_065072245\_15793-model2.5 | -14.714 |
|  | XP\_065072245\_15793-model2.6 | -14.714 |
|  | XP\_065072245\_15793-model2.7 | -14.6989 |
| # cluster 3 - Size: 3 |  |  |
|  | XP\_065072245\_15793-model3 | -14.7096 |
|  | XP\_065072245\_15793-model3.1 | -14.5799 |
|  | XP\_065072245\_15793-model3.2 | -14.411 |
| # cluster 4 - Size: 10 |  |  |
|  | XP\_065072245\_15793-model4 | -14.7088 |
|  | XP\_065072245\_15793-model4.1 | -14.7088 |
|  | XP\_065072245\_15793-model4.2 | -14.7088 |
|  | XP\_065072245\_15793-model4.3 | -14.7088 |
|  | XP\_065072245\_15793-model4.4 | -14.7088 |
|  | XP\_065072245\_15793-model4.5 | -14.7088 |
|  | XP\_065072245\_15793-model4.6 | -14.7088 |
|  | XP\_065072245\_15793-model4.7 | -14.7088 |
|  | XP\_065072245\_15793-model4.8 | -14.6978 |
|  | XP\_065072245\_15793-model4.9 | -14.6978 |
| # cluster 5 - Size: 27 |  |  |
|  | XP\_065072245\_15793-model5 | -14.6761 |
|  | XP\_065072245\_15793-model5.1 | -14.6761 |
|  | XP\_065072245\_15793-model5.2 | -14.6761 |
|  | XP\_065072245\_15793-model5.3 | -14.6743 |
|  | XP\_065072245\_15793-model5.4 | -14.6743 |
|  | XP\_065072245\_15793-model5.5 | -14.6743 |
|  | XP\_065072245\_15793-model5.6 | -14.6743 |
|  | XP\_065072245\_15793-model5.7 | -14.6743 |
|  | XP\_065072245\_15793-model5.8 | -14.6743 |
|  | XP\_065072245\_15793-model5.9 | -14.6743 |
|  | XP\_065072245\_15793-model5.10 | -14.6743 |
|  | XP\_065072245\_15793-model5.11 | -14.6743 |
|  | XP\_065072245\_15793-model5.12 | -14.6743 |
|  | XP\_065072245\_15793-model5.13 | -14.6743 |
|  | XP\_065072245\_15793-model5.14 | -14.6743 |
|  | XP\_065072245\_15793-model5.15 | -14.6743 |
|  | XP\_065072245\_15793-model5.16 | -14.6743 |
|  | XP\_065072245\_15793-model5.17 | -14.6743 |
|  | XP\_065072245\_15793-model5.18 | -14.6743 |
|  | XP\_065072245\_15793-model5.19 | -14.6743 |
|  | XP\_065072245\_15793-model5.20 | -14.6743 |
|  | XP\_065072245\_15793-model5.21 | -14.6743 |
|  | XP\_065072245\_15793-model5.22 | -14.6743 |
|  | XP\_065072245\_15793-model5.23 | -14.6743 |
|  | XP\_065072245\_15793-model5.24 | -14.6743 |
|  | XP\_065072245\_15793-model5.25 | -14.6743 |
|  | XP\_065072245\_15793-model5.26 | -14.6743 |
| # cluster 6 - Size: 4 |  |  |
|  | XP\_065072245\_15793-model6 | -14.6693 |
|  | XP\_065072245\_15793-model6.1 | -14.6693 |
|  | XP\_065072245\_15793-model6.2 | -14.6693 |
|  | XP\_065072245\_15793-model6.3 | -14.6693 |
| # cluster 7 - Size: 6 |  |  |
|  | XP\_065072245\_15793-model7 | -14.6576 |
|  | XP\_065072245\_15793-model7.1 | -14.6576 |
|  | XP\_065072245\_15793-model7.2 | -14.6576 |
|  | XP\_065072245\_15793-model7.3 | -14.6576 |
|  | XP\_065072245\_15793-model7.4 | -14.6314 |
|  | XP\_065072245\_15793-model7.5 | -14.6314 |
| # cluster 8 - Size: 1 |  |  |
|  | XP\_065072245\_15793-model8 | -14.6481 |
| # cluster 9 - Size: 2 |  |  |
|  | XP\_065072245\_15793-model9 | -14.6436 |
|  | XP\_065072245\_15793-model9.1 | -14.4462 |
| # cluster 10 - Size: 4 |  |  |
|  | XP\_065072245\_15793-model10 | -14.609 |
|  | XP\_065072245\_15793-model10.1 | -14.609 |
|  | XP\_065072245\_15793-model10.2 | -14.609 |
|  | XP\_065072245\_15793-model10.3 | -14.609 |
| # cluster 11 - Size: 2 |  |  |
|  | XP\_065072245\_15793-model11 | -14.6036 |
|  | XP\_065072245\_15793-model11.1 | -14.5684 |
| # cluster 12 - Size: 12 |  |  |
|  | XP\_065072245\_15793-model12 | -14.5934 |
|  | XP\_065072245\_15793-model12.1 | -14.5934 |
|  | XP\_065072245\_15793-model12.2 | -14.5934 |
|  | XP\_065072245\_15793-model12.3 | -14.5934 |
|  | XP\_065072245\_15793-model12.4 | -14.5934 |
|  | XP\_065072245\_15793-model12.5 | -14.5934 |
|  | XP\_065072245\_15793-model12.6 | -14.5934 |
|  | XP\_065072245\_15793-model12.7 | -14.5211 |
|  | XP\_065072245\_15793-model12.8 | -14.5211 |
|  | XP\_065072245\_15793-model12.9 | -14.5211 |
|  | XP\_065072245\_15793-model12.10 | -14.5211 |
|  | XP\_065072245\_15793-model12.11 | -14.5211 |
| # cluster 13 - Size: 2 |  |  |
|  | XP\_065072245\_15793-model13 | -14.5848 |
|  | XP\_065072245\_15793-model13.1 | -14.5848 |
| # cluster 14 - Size: 5 |  |  |
|  | XP\_065072245\_15793-model14 | -14.5754 |
|  | XP\_065072245\_15793-model14.1 | -14.5754 |
|  | XP\_065072245\_15793-model14.2 | -14.5349 |
|  | XP\_065072245\_15793-model14.3 | -14.5349 |
|  | XP\_065072245\_15793-model14.4 | -14.5349 |
| # cluster 15 - Size: 2 |  |  |
|  | XP\_065072245\_15793-model15 | -14.5625 |
|  | XP\_065072245\_15793-model15.1 | -14.5365 |
| # cluster 16 - Size: 2 |  |  |
|  | XP\_065072245\_15793-model16 | -14.5419 |
|  | XP\_065072245\_15793-model16.1 | -14.5419 |
| # cluster 17 - Size: 1 |  |  |
|  | XP\_065072245\_15793-model17 | -14.5033 |
|  |  |  |
